# Role of circulating microparticles and cytokines in periodontitis associated with diabetes

**DOI:** 10.3389/fmed.2024.1394300

**Published:** 2024-08-26

**Authors:** Bárbara Adelungue Cassiano, Ana Luíza Pereira Assunção Silveira, Yeon Jung Kim, Jônatas Bussador do Amaral, Luiz Henrique da Silva Nali, André Luis Lacerda Bachi, Leonardo Diniz Resende, Francisco Antonio Helfenstein Fonseca, Maria Cristina de Oliveira Izar, Izabela Dorota Tuleta, Jefferson Russo Victor, Débora Pallos, Carolina Nunes França

**Affiliations:** ^1^Health Sciences Post Graduation, Santo Amaro University, São Paulo, Brazil; ^2^Odontology Post Graduation, Santo Amaro University, São Paulo, Brazil; ^3^ENT Research Laboratory, Otorhinolaryngology-Head and Neck Surgery Department, Federal University of São Paulo, São Paulo, Brazil; ^4^Cardiology Division, Department of Medicine, Federal University of São Paulo, São Paulo, Brazil; ^5^Department of Medicine-Cardiology, Albert Einstein College of Medicine, New York, NY, United States

**Keywords:** microparticles, IL-6, IL-10, periodontitis, diabetes, cytokines, non-surgical periodontal treatment, systemic inflammation

## Abstract

**Background:**

Periodontitis is a chronic inflammatory condition that affects the supporting tissues of the teeth, and can lead to serious complications such as tooth loss and systemic health problems, including diabetes, which have a bidirectional relationship with periodontitis. Circulating microparticles originate from different cell types after stimuli such as activation or apoptosis. Interleukins are related to processes in the regulation of the immune response, inflammation, and cell growth. This study aimed to evaluate circulating microparticles as well as interleukins in the plasma, at baseline and 1 month after the end of the non-surgical periodontal treatment.

**Methods:**

Samples were collected from 45 patients, with moderate to severe periodontitis with diabetes (*N* = 25) and without diabetes (*N* = 20). Microparticles were evaluated in the platelet-poor plasma by flow cytometer. Cytokine levels were evaluated by the enzyme immunoabsorption assay (ELISA).

**Results:**

Higher levels of the pro-inflammatory cytokines were found in the group with diabetes compared to the non-diabetic group both at baseline and 1 month after the end of the treatment. A higher IL-6/IL-10 ratio was found in patients with diabetes compared to the group without diabetes at T0 and T1, whereas an increased IFN-γ/IL-10 ratio was only found at T1 in patients with diabetes in comparison to the group without diabetes. In the group with diabetes, it was verified positive correlations between IL-10 and IL-6 or IFN-γ and a negative correlation between IL-6 and PMP, at T0; in contrast, in the T1, negative correlations were found between TNF-α and IL-10 or PMP. Besides, at T0, it was evidenced positive correlations both between circulating TNF-α and IL-6, and IL-10 and EMP, as well as a negative correlation between IL-10 and PMP in the group with diabetes. In addition, it was observed in T1 positive correlations between levels of TNF-α and IL-6, IFN-γ, or IL-10, and between PMP and IFN-γ, and between EMP and IL-6, TNF-α and IFN-γ in this group.

**Conclusion:**

The results suggest a modulatory effect of the periodontitis associated with diabetes, as well as the periodontal treatment, in the systemic inflammatory status of the participants of the study.

## Introduction

Periodontitis is one of the most common causes of tooth loss in adults (1), defined as a chronic inflammatory disease that affects tooth protection and insertion structures, of multifactorial origin, and is associated with the accumulation of dysbiotic biofilm that contains anaerobic pathogens, being considered the initial etiological factor of periodontitis (1–3). Biofilm consists of a granular film composed of bacteria and their products, desquamated cells, and extracellular polymer and can develop more easily on surfaces close to the neck, which can lead to tissue damage and, sometimes, periodontal rupture (4). Studies have shown a direct relationship between the amount of accumulated biofilm, level of intensity, and tissue destruction (5, 6).

Periodontitis is one of the main diseases of the oral cavity that has a high prevalence rate and global involvement, occupying the second largest cause of dental disease in the human population, being more frequent in adults, varying in different regions of the world (7). The prevalence of the severe form of periodontitis in the world is 11% (8).

In recent decades, periodontitis has been associated with the appearance of systemic disorders, including cardiovascular diseases and diabetes (9). The pathogens present in the biofilm destroy the periodontal pocket epithelium, allowing harmful endotoxins and exotoxins to enter the bloodstream, leading to bacterial dissemination and systemic infection, consequently having an increased inflammatory response (10), with oxidative stress, greater recruitment of leukocytes, increased production of inflammatory mediators and apoptosis (11, 12).

Periodontitis is the most common cause of chronic inflammation in patients with diabetes. Studies show the relationship between the destruction of periodontal tissue and the severity of insulin resistance, where diabetes is a predisposing factor for periodontitis and periodontitis makes metabolic control difficult in patients with diabetes, showing a clear relationship between the diseases (1, 11, 13). Both diseases cause microvascular changes in the crevicular fluid, as the inflammation associated with periodontitis is not limited to the gingival tissue, but can also trigger a systemic inflammatory response causing a disturbance in metabolism and alteration of host immune response and this association between the two diseases has been reported by studies since the 1960s, both of which are associated with a pro-inflammatory state, with oxidative stress, greater recruitment of leukocytes, increased production of inflammatory mediators and apoptosis (11, 12).

Microparticles (MP) are a population of vesicles released by different cells, known to carry content from their cell of origin, such as mRNA, lipoproteins, and cytokines, among others. The release of MP occurs after apoptosis or activation by different stimuli, such as lipopolysaccharide (LPS) from Gram-negative bacteria and cytokines such as tumor necrosis factor alpha (TNF-α), interleukin 6 (IL-6) and interleukin 1 (IL-1β) (14–16).

The increased presence of MP in the gingival crevicular fluid is associated with the local inflammatory response, cellular activation, and the release of these extracellular vesicles. These interactions can lead to the dissemination of MP into the circulatory system, contributing to their increase in plasma. Circulating MPs can penetrate cells, transfer antigens, affect cellular gene expression, and activate cell signaling pathways that are associated with proliferation, survival, adhesion, and chemotaxis (16).

Following the literature, pro-and anti-inflammatory cytokines play an important role in the development and progression of both diabetes and periodontitis. In this sense, it has been reported not only that elevated systemic levels of IL-6 and TNF-α in the simultaneous presence of diabetes and periodontitis than in periodontitis alone, as well as the increased levels of these well-known pro-inflammatory cytokines in GCF can activate osteoclasts, which promote bone resorption, destruction of alveolar bone, tissue inflammation, and periodontitis progression (17–19). Moreover, in patients with type 2 diabetes the circulating IFN-γ levels, one of the main proinflammatory cytokines from T helper (Th)-1 cells, are increased in comparison to healthy control individuals (20, 21), which reinforces the presence of a pro-inflammatory state in this disease (22). Concerning periodontitis, whilst higher IFN-γ levels in GCF were associated with periodontitis progression (23), this cytokine can also play a protective role depending on the stage of the disease and the immunological context. Beyond these pro-inflammatory cytokines, the classical anti-inflammatory cytokine IL-10 has been also studied in the diabetes and periodontitis context (24), and, in general, it was reported that both patients with periodontitis and type-2 diabetes and patients with periodontitis but not diabetics presented lower IL-10 levels than in control groups (24–26). It is noteworthy to mention that IL-10 presents a corollary action in modulating the immune response in these diseases since it is broadly accepted that IL-10 can control or prevent TNF-α and IL-6 production, thus modulating inflammation (26, 27).

Since there is a scarcity of studies showing the effect of non-surgical periodontal treatment on MP and cytokines levels, the objective of this study is to evaluate the effect of non-surgical periodontal treatment concerning circulating MP and cytokines, in the presence or not of diabetes.

## Methods

This is a case-control study, based on a clinical trial (28) that included patients being monitored at the Dentistry Clinic of Universidade Santo Amaro—UNISA, aged between 40 and 75 years, of both sexes, diagnosed with moderate and advanced periodontitis, associated or not with diabetes.

Data such as risk factors, clinical examinations, and blood sample collection were obtained through clinical visits, at baseline and 1 month after the end of non-surgical periodontal treatment.

Patients read and signed the informed consent form (ICF). The study was registered as a clinical trial before its start (ReBEC RBR-35szwc). As inclusion criteria: patients with periodontitis (moderate or severe), diagnosed by two periodontists and who had at least 10 teeth in the oral cavity; as exclusion criteria: to be toothless, having received periodontal treatment in the last 6 months, being a smoker, having gingivitis or mild periodontitis.

Patients were separated into two groups: periodontitis with diabetes and periodontitis without diabetes. The presence of diabetes mellitus was determined through:

1. Glycated hemoglobin (HbA1c) ≥6.5%.
2. Blood glucose ≥200 mg associated with symptoms such as weight loss, polydipsia/polyuria.
3. Use of antidiabetic medications with a history of previous diagnosis.

The patients were clinically evaluated by two previously trained periodontists; 10% of the sample was examined twice for each of the clinical criteria evaluated, to obtain intra-examiner diagnostic reliability measured by the Kappa statistic (between 0.8 and 1.0).

The probing depth (PD) was measured in all sites, using a Williams-type manual periodontal probe, being measured from the free gingival margin to the base of the periodontal pocket.

The clinical attachment level (NCI) was obtained from all sites examined by measuring the distance from the enamel-cementum junction (ECJ) to the gingival margin (MG) adding to the PD measurement. In other words: NCI = PD + (JEC to MG).

Gingival bleeding was noted dichotomously 20 s after obtaining the periodontal probing. The gingival condition of the subjects was assessed using the gingival index. Oral hygiene was assessed using the plaque index.

After clinical periodontal examination, individuals were diagnosed according to the classification of periodontal diseases, and individuals diagnosed with stage II, III, and IV periodontitis were included. After oral hygiene instruction for the patients, the sites diagnosed with periodontitis were subjected to non-surgical periodontal treatment, which consisted of oral hygiene instruction, supra and subgingival scaling, root planning, and dental polishing.

Concerning the phenotypic characterization of circulating MP, the peripheral blood was collected from patients in blood tubes containing the anticoagulant citrate and submitted to centrifugation at 160 g, 22°C for 10 min to obtain platelet-rich plasma (PRP). Following, the PRP was then centrifuged at 1,500 g, 22°C for 6 min, to obtain platelet-poor plasma (PPP). Next, the PPP (70 mL) was incubated for 20 min at room temperature with anti-CD105 conjugated to APC, anti-CD42 conjugated to FITC combined with anti-CD31 conjugated to PE, and anti-CD14, to identify endothelial, platelet and monocytic microparticles, respectively. The isotypes IgG1 FITC, IgG1 PE, and IgG1 APC (BD Biosciences, Franklin Lakes, NJ, United States) were used as controls. After incubation, 300 mL of PBS was added and immediately read on a flow cytometer (FACSCalibur—BD Biosciences, Franklin Lakes, NJ, United States), approximately 30,000 events were acquired.

Circulating levels (plasma or serum) of total protein, albumin, creatinine, iron, uric acid, glucose, D-fructosamine, triglycerides, and total cholesterol and fractions (LDL and HDL) were determined using commercial kits (Bioclin-Quibasa, Belo Horizonte, Minas Gerais, Brazil), following manufacturer’s instruction, in a semi-automatized system (Dimension^®^ RxL Max^®^ Integrated Chemistry System, Siemens, Deerfield, IL, United States).

The circulating levels of cytokines IL-6, IL-10, TNF-α, and IFN-γ were determined by using the commercial ELISA kits (Invitrogen), following the manufacturer’s recommendations for each kit. Briefly, the 96-well plate was coated with capture antibody overnight at 4°C. After three washes, 200 μL of ELISA/ELISPOT Diluent (1X) was added and incubated at room temperature for 1 h, then the wells were washed at least once with wash buffer and 100 μL of samples (plasma) were added to the appropriate wells and plate was sealed and incubated for 2 h. Following three more washes, 100 μL of diluted detection antibody was added to the wells, sealed, and incubated for another 1 h. After incubation, another wash was carried out and 100 μL of diluted streptavidin-HRP was added and incubated for 30 min. After another wash, 100 μL of 1X TMB solution was added to the wells, at room temperature, and after 15 min the reaction was stopped with the addition of 100 μL of stop solution. The plate was read at 450 nm in an ELISA reader.

### Statistical analysis

All data obtained in the volunteer groups were initially evaluated to verify their normality by using the Shapiro–Wilk test and the homogeneity of variance was assessed by the Levene test. Based on it, parametric variables were presented as mean and standard deviation (X ± SD) and were statistically analyzed using the paired *t*-test or one-way ANOVA with Tukey’s post-hoc test, whereas non-parametric variables were presented as a median and interquartile range and were statistically analyzed using the Wilcoxon test (comparisons between visits, in each group) or Kruskal–Wallis test (comparisons between groups) with Dunn’s post-hoc test. In addition, Pearson’s or Spearman’s rank correlation coefficient analysis was used to evaluate the association between the parameters assessed here. The SPSS version 18.0 program was used and a significance level of *p* < 0.05 was considered.

## Results

**Figure 1:**
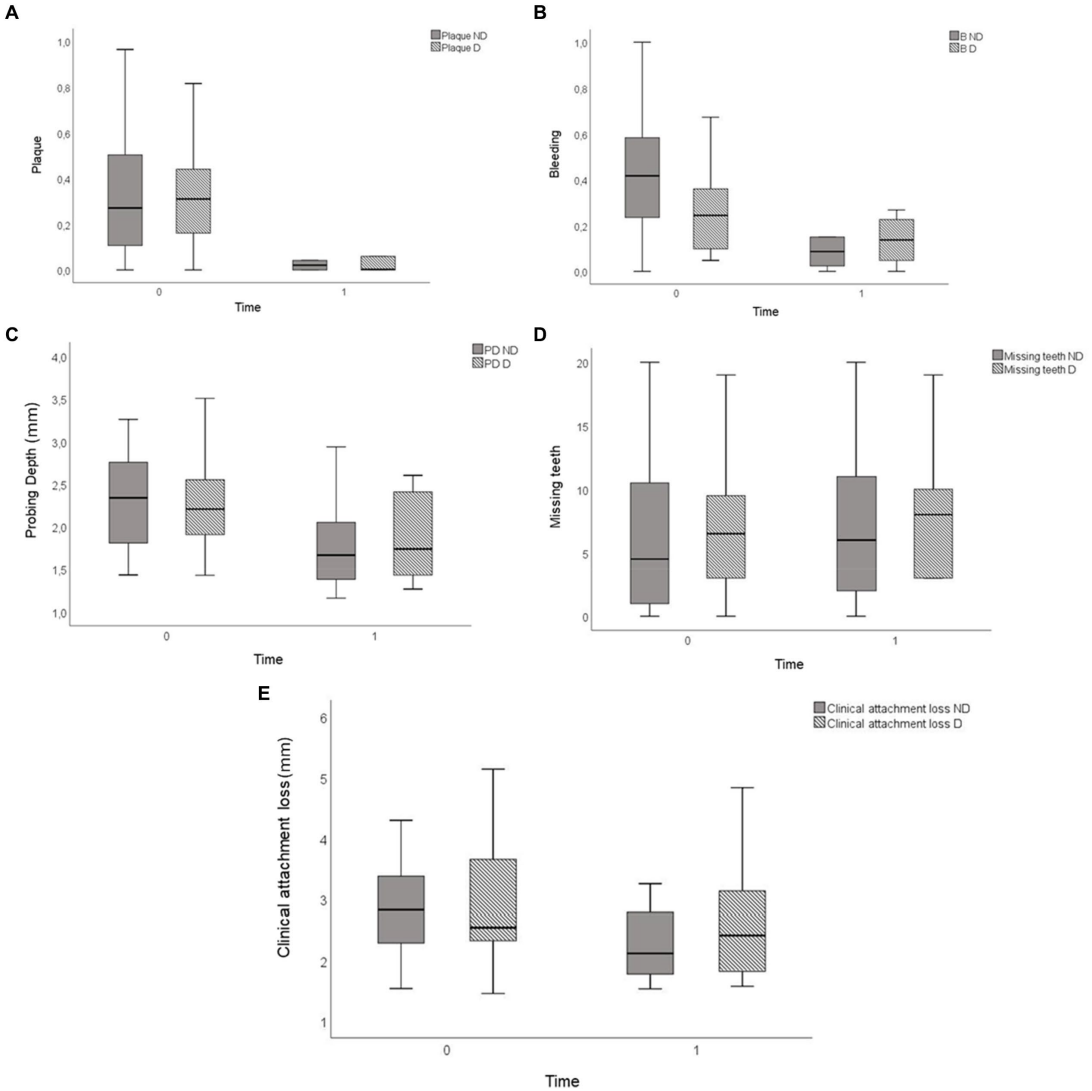
Periodontal clinical data from the participants of the study. **(A)** Plaque. **(B)** Bleeding. **(C)** Probing depth. **(D)** Missing teeth. **(E)** Clinical attachment loss.

The participants (*n* = 45) were classified as having moderate to severe periodontitis with diabetes (*N* = 25) and without diabetes (*N* = 20), presenting median age (interquartile range—IQR) of 55 (44–61) years, being 19 men (42%) and 26 women (58%). The periodontal clinical characteristics are represented in Figure 1.

The results concerning biochemical parameters are shown in Table 1, at baseline and 1 month after the end of the periodontal treatment.

**Table 1:** Biochemical parameters of the participants of the study, *n* = 45.

Metabolite	Baseline	One month	*p*
Uric acid (mg/dL), mean (SD)	6.61 (1.77)	7.19 (1.10)	0.37^a^
Albumin (g/dL), mean (SD)	3.19 (0.71)	3.41 (0.55)	0.31^a^
Serum creatinine (mg/dL), median (IR)	0.47 (0.18–0.85)	0.65 (0.25–0.82)	0.53^b^
Serum iron (μg/dL), median (IR)	74.95 (37.38–128.16)	73.06 (51.19–120.29)	0.86^b^
Fructosamine (μmol/L), mean (SD)	269.39 (146.67)	345.12 (186.55)	0.13^a^
Glucose (mg/dL), mean (SD)	126.64 (142.58)	142.58 (59.67)	0.37^a^
HDL-C (mg/dL), mean (SD)	35.67 (2.77)	35.50 (2.03)	0.65^a^
Total cholesterol (mg/dL), mean (SD)	122.27 (19.28)	121.69 (28.21)	0.80^a^
Triglycerides (mg/dL), mean (SD)	97.93 (20.96)	102.13 (27.90)	0.60^a^

a Paired *t*-test.

b Wilcoxon test.

Table 2 shows the percentages of circulating MP in the PPP of all participants. There were no differences in the three MP populations when comparing baseline and 1 month after the end of periodontal treatment.

**Table 2:** Percentage of circulating microparticles in platelet-poor plasma, comparisons between visits.

Microparticles	Baseline	One month	*p* intra groups
Endothelial	0.40 (0.10)	0.19 (0.05)	0.30
Platelet	66.45 (3.52)	62.82 (5.15)	0.08
Monocytic	6.06 (0.67)	6.63 (1.03)	0.88

The data represent the means (standard errors of the means—SEM). Wilcoxon test, intra-group comparisons (baseline × 1 month after the end of treatment).

Table 3 shows the circulating levels of MP in each group, compared separately, before and 1 month after the end of treatment. Neither significant differences were found in the values observed in each volunteer group at baseline and 1 month after the end of treatment nor when comparing both volunteer groups.

**Table 3:** Percentage of circulating microparticles in platelet-poor plasma.

Microparticles	With diabetes		*p* intra groups	Without diabetes		*p* intra groups	*p* between groups	
	Baseline	One month		Baseline	One month		Baseline	One month
Endothelial	0.57 (0.18)	0.20 (0.06)	0.15	0.24 (0.06)	0.18 (0.11)	0.83	0.43	0.56
Platelet	65.81 (5.95)	67.38 (6.45)	0.38	68.42 (3.83)	58.99 (8.98)	0.14	0.68	0.49
Monocytic	6.57 (1.06)	6.14 (1.04)	0.69	5.59 (0.86)	7.64 (2.07)	0.88	0.57	0.93

Comparisons within groups (baseline and after treatment for each group) and between groups (with and without diabetes). The data is represented as means (standard errors of the means—SEM). Wilcoxon test, intra-group comparison (baseline × final in each group). Mann–Whitney test, comparisons between groups (with and without diabetes for each visit).

**Figure 2:**
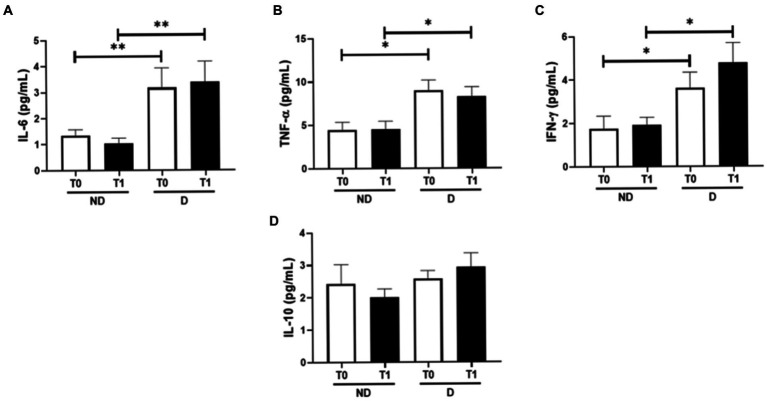
Plasma concentration of the pro-inflammatory interleukins IL-6 **(A)**, TNF-α **(B)**, IFN-γ **(C)**, and the anti-inflammatory interleukin IL-10 **(D)**. ^*^p < 0.05 and ^**^p < 0.01.

Figure 2 shows the systemic levels of cytokines IL-6 (A), TNF-α (B), IFN-γ (C), and IL-10 (D) both at baseline (T0) and 1 month after the end of the treatment (T1) in the volunteers who presented periodontitis in conjunction or not with diabetes. Higher circulating levels of pro-inflammatory cytokines (IL-6—A, TNF-α—B, and IFN-γ—C) were found in the group of patients with periodontitis and diabetes than in the non-diabetic group both at baseline and 1 month after the end of the treatment (IL-6, *p* = 0.0098 at T0 and *p* = 0.0061 at T1; TNF-α, *p* = 0.012 at T0 and *p* = 0.022 at T1; and IFN-γ, *p* = 0.032 at T0 and *p* = 0.029 at T1). No differences were found in the IL-10 levels.

**Figure 3:**
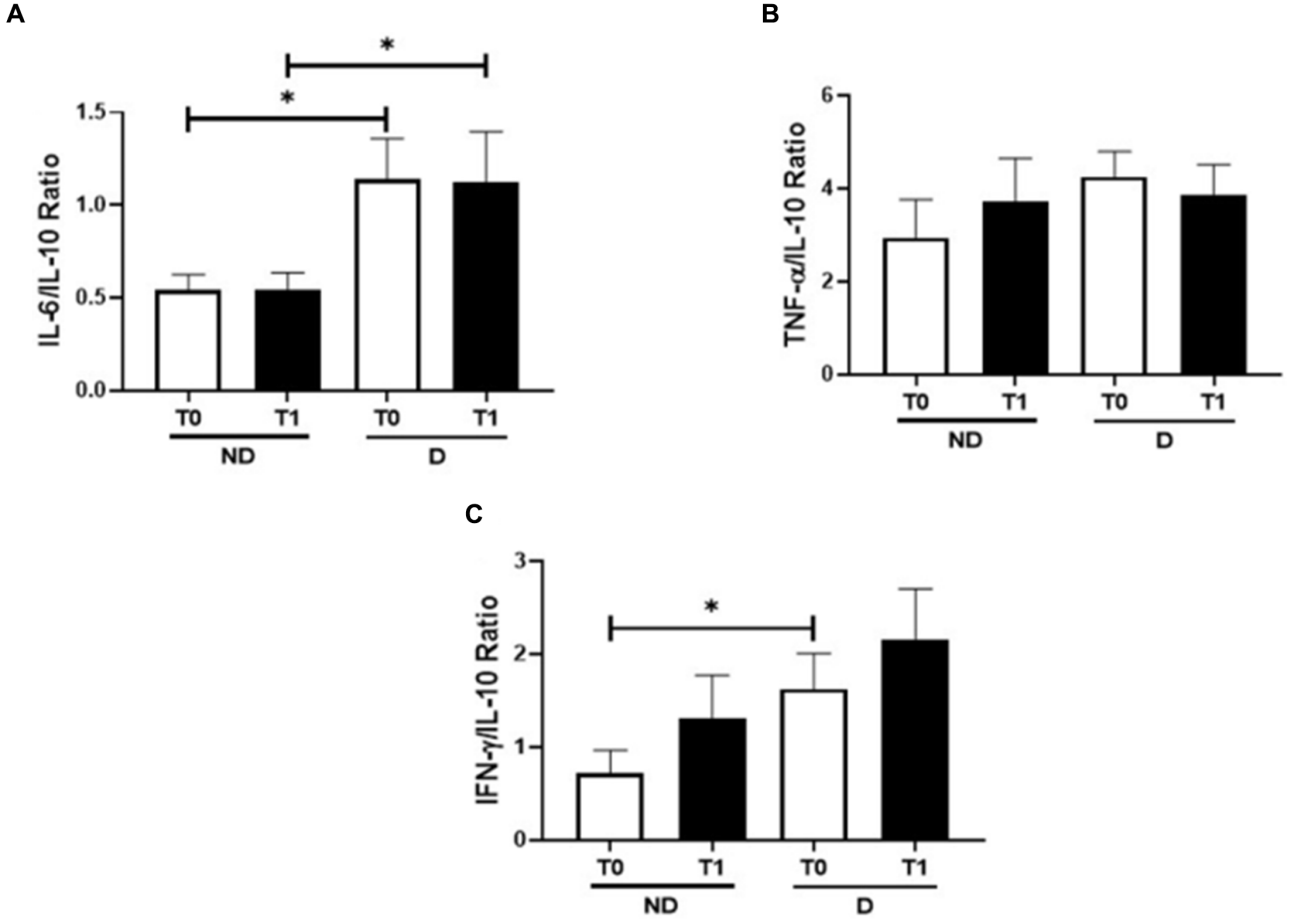
Modulation of the pro-inflammatory and anti-inflammatory interleukins, IL-6/IL-10 **(A)**, TNF-α/IL-10 **(B)**, and IFN-γ/IL-10 **(C)**. ^*^p < 0.05.

Figure 3 shows the ratios between IL-6/IL10 (Figure 2A), TNF-α/IL-10 (Figure 2B), and IFN-γ/IL-10 (Figure 2C) in the volunteer groups both before and after the periodontitis treatment. A higher IL-6/IL-10 ratio was found in patients with diabetes than the values observed in the group without diabetes at T0 (*p* = 0.029) and T1 (*p* = 0.025), whereas an increased IFN-γ/IL-10 ratio was only found at T1 in patients with diabetes compared to the group without diabetes (*p* = 0.041). Regarding the TNF-α/IL-10 ratio, there was no significant difference.

Table 4 shows the correlation analysis between the circulating levels of MP and cytokines evaluated in the present study. Concerning the group of patients without diabetes (Table 4) it was verified not only positive significant correlations between levels of IL-10 and IL-6 or IFN-γ but also a negative significant correlation between the levels of IL-6 and PMP, at baseline (T0). In contrast, after the treatment (T1) negative significant correlations were found between levels of TNF-α and IL-10 or PMP. Regarding the group with diabetes (Table 4), at baseline (T0), it was evidenced positive significant correlations both between circulating levels of TNF-α and IL-6, and between levels of IL-10 and EMP, as well as a negative significant correlation between IL-10 and PMP. In addition, 30 days after the end of treatment (T1), it was observed positive significant correlations between levels of TNF-α and IL-6, IFN-γ, or IL-10, and also between PMP and IFN-γ, as well as between EMP and IL-6, TNF-α and IFN-γ.

**Table 4:** Correlations between microparticles and interleukins in the groups, in both visits (T0 and T1).

Group—periodontitis without diabetes					
Parameters	Baseline (T0)		Parameters	30 days after (T0)	
	rho-value	*p*-value		rho-value	*p*-value
IL-10 × IFN-γ	0.713	**0.004** ^*^	TNF-α × IL-10	0.422	−0.701
IL-10 × IL-6	0.569	**0.036** ^*^	TNF-α × PMP	0.424	−0.7
IL-6 × PMP	−0.608	**0.021** ^*^			

Group—periodontitis with diabetes					
Parameters	Baseline (T0)		Parameters	30 days after (T1)	
	rho-value	*p*-value		rho-value	*p*-value
IL-6 × TNF-α	0.504	**0.016** ^*^	IL-6 × TNF-α	0.561	**0.025** ^*^
IL-10 × EMP	0.601	**0.003** ^*^	TNF-α × IFN-γ	0.697	**0.003** ^*^
IL-10 × PMP	−0.468	**0.027** ^*^	TNF-α × IL-10	0.718	**0.002** ^*^
			TNF-α × EMP	0.691	**0.005** ^*^
			IL-6 × EMP	0.534	**0.040** ^*^
			IFN-γ × EMP	0.582	**0.025** ^*^
			IFN-γ × MMP	0.589	**0.023** ^*^

The data above represent the positive and negative correlations between circulating interleukins and microparticles in the both groups, at baseline visits (T0) and after 1 month of the treatment (T1). IL-6, interleukin 6; TNF-α, tumor necrosis factor alpha; IFN-γ, interferon gamma; IL-10, interleukin 10; MMP, monocytic microparticles; EMP, endothelial microparticles; PMP, platelet microparticles. ^*^p < 0.05.

## Discussion

The present study aimed to evaluate the effect of non-surgical periodontal treatment in individuals with or without diabetes on the circulating MP levels and the inflammatory status. In this sense, although the lack of significant alterations in the inflammatory status was not a novelty, for the first time, it was evidence that the MP levels, regardless of the type, did not show significant differences 30 days after the end of the treatment in both volunteer groups. Furthermore, the findings obtained in the correlation analyses can also improve our understanding of the effect of non-surgical periodontal treatment, in a short time, in the volunteer groups participants here.

According to the literature, associations between MP and inflammatory responses occur for several reasons. For instance, activated cells, such as platelets, endothelial cells, and leukocytes, are induced to release MP as part of their response to the inflammatory stimulus. Interestingly, the MP carries a variety of molecules including some cytokines, such as IL-6, and TNF-α, among others, which can show the occurrence of a mutual interplay between these molecules (29, 30). Based on it, our findings corroborate this association since it was evidenced significant correlations between the MP and cytokines assessed here, particularly in the volunteer group who presented diabetes.

In this respect, it was verified that, at baseline, the group without diabetes showed a negative correlation between circulating levels of IL-6 and PMP, which was an unexpected finding since a strongly positive correlation between these molecules has been reported (31, 32), whereas the group with diabetes showed that the circulating IL-10 levels were negatively correlated with PMP and also positively correlated with EMP.

In agreement with the literature, the systemic elevations of EMP are closely related to endothelial damage and/or apoptosis, which can serve as a remarkable biomarker in both damage and repair of endothelial tissue, including in diabetic individuals (33), the PMP, particularly generate from platelets in circulation, presents not only an important role in cell communication, angiogenesis, homeostasis, and coagulation but also a strong association with many diseases, such as diabetes (32, 34). Thus, despite the positive correlation between IL-10 and EMP can putatively demonstrate that a regulatory mechanism was present in the diabetic group, due to increased IL-10 levels have been related to an improvement of endothelial function in people presenting systemic inflammation (35), the negative correlation between IL-10 and PMP can show that this regulatory state was not completely achieved.

Based on this, we can suppose that periodontitis could impact these findings, maybe leading to an impairment of the regulatory state, since it was detected PMP in gingival crevicular fluid from individuals with diabetes and severe periodontitis (36), which shows that this molecule can be bioavailable in different sites, both in the oral cavity and systemically. Although we cannot confirm, the occurrence of periodontitis could putatively be involved in the negative correlation between IL-6 and PMP in the non-diabetic group, which led to this unexpected association between these molecules.

Both corroborating this suggestion and following the literature, the results obtained 30 days after the end of the periodontitis treatment showed only positive significant correlations between the circulating levels of pro-inflammatory cytokines and MPs. Interestingly, whereas in the non-diabetic group, we observed only a positive significant correlation between TNF-α and PMP, which is in agreement with the literature (37–39), in the diabetic group we verified several positive significant correlations, in fact, these positive correlations between EMP and TNF-α, IFN-γ, and IL-6 were already reported (40–42), including in diabetic individuals (43), whereas the positive correlation between IFN-γ and MMP it has been not reported in the literature, but is aligned to the association between the pro-inflammatory cytokines and MPs. Taking these data together, the modulatory effect, in a short time, of peridontitis treatment in the volunteer groups is a novelty of the study and can reinforce the observation that this oral disease affects systemic inflammatory status.

Beyond these results, it is utmost of importance to mention that other positive significant correlations were observed between the systemic levels of the cytokines assessed here in both volunteer groups at baseline and post-treatment. Based on the data evidenced in the group non-diabetic, in which the circulating IL-10 levels were positively correlated with IL-6 and IFN-γ, at baseline, as well as with TNF-α after the treatment ends, we can suggest that a regulatory status was maintained even with the presence of periodontitis. On the other hand, in the group with diabetic individuals, the most of positive correlations found at baseline and post-treatment was between the pro-inflammatory cytokines, which reinforced not only the pro-inflammatory feature of diabetes (44) but also our suggestion that 30 days after the end of the periodontitis treatment was not able to promote remarkable alterations in the systemic inflammatory status in those individuals.

In addition to these findings, the results obtained in the evaluation of the ratio between the pro-inflammatory cytokines and the anti-inflammatory cytokine IL-10, carried out here, allowed us to verify the modulatory effect of the periodontitis, as well as its treatment, in the systemic inflammatory status in the volunteer groups. In this sense, even though the TNF-α/IL-10 ratio did not show a difference between the volunteer group, the higher IL-6/IL-10 ratio found in the group with diabetes, at baseline (T0) and post-treatment (T1), in conjunction with the higher IFN-γ/IL-10 ratio found at baseline (T0), than the values observed in the group without diabetes indicates that a prominent pro-inflammatory status was present in the diabetic individuals. It is paramount to highlight that the ratio assessment has been considered as an accurate measure concerning the balance of pro-and anti-inflammatory cytokines in different contexts, including in diabetes (33). Although these data, again, can reinforce the pro-inflammatory feature of diabetes, the lack of significant difference in the IFN-γ/IL-10 ratio 30 days after the periodontitis treatment ends (T1) can demonstrate that this procedure was able to impact this ratio, apparently in the non-diabetic group.

Corroborating the idea that the ratio of pro-and anti-inflammatory cytokines can be useful to improve our understanding of its balance in the study context, the observation isolated of higher circulating levels of the pro-inflammatory cytokines IL-6, TNF-α, and IFN-γ in the diabetic volunteers than in individuals without this disease, at baseline (T0) and post-treatment (T1), not only were expected but also were not able to show any effect of peridontitis treatment in these volunteer groups.

Regarding non-surgical periodontal treatment applied in the present study, it is worth pointing out that it remains the gold standard for managing chronic periodontitis, due to its capacity to promote subgingival scaling and root planning, as well as to remove supragingival plaque. Moreover, the association between this therapy and adjunctive measures can improve its efficacy leading to a satisfactory elimination of biofilm and, consequently, restoring a balanced microbiota environment in the periodontal site (45). Therefore, non-surgical therapy is useful for decreasing pocket depth, favors the recovery of clinical attachment through the restoration of highly perfused and collagen-rich connective tissues, as well as reduces the local inflammatory stimulation (46). This last prominent non-surgical therapy effect is very important since maintaining the chronic pro-inflammatory status associated with periodontitis is involved in the limited capacity to repair periodontal tissue following inflammatory insults, especially in diabetic individuals (47). In addition, diabetic patients present a higher loss of fibroblasts and osteoblasts by apoptosis, which can favor the limited repair of injured tissue (48). Although we cannot affirm, based on these pieces of information, we can suggest that the therapy used in the present study was able to promote the reduction of inflammation in the periodontal site, and, as expected, the presence of diabetes had a pivotal role in the findings related to the cytokines and MP found in the volunteer groups.

## Data Availability

The original contributions presented in the study are included in the article/supplementary material, further inquiries can be directed to the corresponding author.
